# Association Between the “Weekend Warrior” Physical Activity Pattern and Musculoskeletal Health Outcomes: A Systematic Review

**DOI:** 10.3390/healthcare14213642

**Published:** 2026-10-27

**Authors:** Chen Hong, Xiaoyou Zhang, Qi Li, Yang Yang, Shiyu Wang, Wenrui Zhao, Boyi Zong

**Affiliations:** 1 School of Physical Education, Hubei University, Wuhan 430062, China; 202421109011964@stu.hubu.edu.cn (C.H.); 202421109011959@stu.hubu.edu.cn (Y.Y.); 2 School of Physical Education, Central China Normal University, Wuhan 430079, China; lqccnu123@mails.ccnu.edu.cn; 3 College of Physical Education and Health, East China Normal University, Shanghai 200241, China; 52261000010@stu.ecnu.edu.cn; 4 Key Laboratory of Adolescent Health Assessment and Exercise Intervention of Ministry of Education, East China Normal University, Shanghai 200241, China; 5 College of Physical Education and Health Sciences, Zhejiang Normal University, Jinhua 321004, China; zhaowenrui108@zjnu.edu.cn; 6 School of Sport Sciences, Nanjing Normal University, Nanjing 210023, China; 12211@njnu.edu.cn

**Keywords:** weekend warrior, physical activity pattern, musculoskeletal health, bone mineral density, osteoporosis, sarcopenia, osteoarthritis

## Abstract

**Background:** Musculoskeletal conditions impose an increasing health burden, particularly in ageing populations. For adults who find it difficult to exercise throughout the week, completing the recommended amount of moderate-to-vigorous physical activity (MVPA) over only 1–2 days, commonly described as the “weekend warrior” (WW) pattern, may offer a more practical approach. Whether this concentrated pattern is associated with musculoskeletal outcomes comparable to those observed with regularly distributed activity remains uncertain. This systematic review synthesized current evidence concerning the relationships of the WW pattern with musculoskeletal outcomes and examined how these associations compared with those observed in inactive (IA) and regularly active (RA) adults. **Methods:** PubMed, Web of Science, and Scopus were searched from database inception through 30 June 2026, following PRISMA recommendations. Ten observational investigations, including four cohort and six cross-sectional studies, were eligible, with individual sample sizes ranging from 3539 to 83,376 adults. Cohort studies were evaluated using the Newcastle–Ottawa Scale, whereas cross-sectional studies were appraised with the Agency for Healthcare Research and Quality checklist. **Results:** Across the included studies, adults following a WW pattern generally had more favorable estimates for low bone mineral density (BMD), osteoporosis, osteoarthritis, spinal degenerative changes, inflammatory arthritis, and sarcopenia than inactive adults. Where WW and RA patterns were directly contrasted, their associations with most musculoskeletal outcomes were broadly similar, although WW was associated with a higher hazard of osteoarthritis in one study, a lower hazard of fractures in another, and lower odds of sarcopenia among WW participants. Subgroup analyses further suggested that these associations may vary across population characteristics, although the patterns were not uniform. Associations with BMD tended to be more pronounced in men and in participants with body mass index (BMI) < 25 kg/m^2^. Findings for osteoporosis varied according to age and genetic susceptibility, while age- and sex-specific associations with sarcopenia differed across studies. One exploratory analysis also suggested that the association with osteoporosis may vary according to the timing of MVPA across the day. **Conclusions:** Meeting the recommended weekly amount of MVPA over 1–2 days was generally associated with more favorable musculoskeletal outcomes than inactivity, while the observed estimates were often close to those reported for regularly distributed activity. These findings support the possibility that achieving sufficient weekly activity may remain relevant even when exercise is concentrated into fewer days. Nevertheless, the evidence is entirely observational and is limited by heterogeneous definitions of WW, differences in physical activity assessment, residual confounding, overlapping study populations, and insufficient investigation of injury outcomes. Current findings therefore cannot establish causality or demonstrate equivalence or non-inferiority between WW and RA patterns.

## 1. Introduction

With the acceleration of global population ageing and continued increases in life expectancy, the burden of degenerative musculoskeletal conditions, including sarcopenia, osteoporosis, and osteoarthritis, is rising rapidly worldwide [1,2,3,4,5]. The growing burden of these conditions represents a major public health challenges facing ageing societies in the 21st century [6].

Since 1990, the number of people worldwide living with conditions that could benefit from rehabilitation has increased by more than 63%, with musculoskeletal conditions accounting for a particularly substantial burden and affecting more than 1.71 billion people globally [1]. Progressive deterioration of the musculoskeletal system can impose considerable physical and psychological burdens on affected individuals [7], while older adults may experience chronic pain, disability, and loss of independence and are also at increased risk of falls, fractures, and prolonged immobility, which may further contribute to serious complications [7,8], ultimately reducing quality of life and placing a substantial socioeconomic burden on healthcare systems worldwide [4,8].

Physical activity (PA) represents an important and cost-effective non-pharmacological strategy for maintaining musculoskeletal health and for preventing and managing a range of musculoskeletal disorders [9,10,11]. In addition, weight-bearing and resistance exercises can stimulate bone formation and reduce bone loss [12,13], while maintaining or improving muscle mass and strength [14] and enhancing joint function [15]. Although the health benefits of PA are well documented, an estimated 27.5% of the global adult population remains insufficiently active in 2016, according to World Health Organization recommendations [16]. Therefore, identifying effective and feasible strategies to reduce the risk of musculoskeletal disorders remains an urgent public health priority.

Current World Health Organization recommendations advise adults to accumulate 150–300 min of moderate-intensity physical activity (MPA) or 75–150 min of vigorous-intensity physical activity (VPA) each week, to obtain health benefits, including benefits for musculoskeletal health [17]. However, occupational demands, family responsibilities, and other competing commitments may make it difficult for time-constrained individuals to engage consistently in RA. In this context, Lee et al. [18] first introduced the “weekend warrior” (WW) pattern in 2004, referring to the accumulation of the recommended weekly volume of moderate-to-vigorous physical activity (MVPA) within 1–2 days. This pattern has attracted renewed attention because accumulating evidence suggests that it may be associated with health outcomes comparable to those observed with more regularly distributed PA [19,20,21].

Existing studies generally classify individuals into three PA patterns based on current guideline thresholds: inactive (IA), defined as engaging in <150 min of MVPA per week; regularly active (RA), defined as engaging in ≥150 min of MVPA per week but not meeting the criteria for WW; and WW, defined as engaging in ≥150 min of MVPA per week, with at least 50% of the total weekly MVPA accumulated within 1–2 days.

Although a substantial body of research has demonstrated the importance of PA for maintaining bone and muscle health, most existing evidence has focused on the traditional RA, whereas research specifically examining the WW pattern remains relatively limited. Moreover, evidence on the WW pattern has predominantly focused on cardiometabolic health and mortality outcomes, with considerably less attention given to musculoskeletal health. At present, there is insufficient evidence to determine whether the WW pattern is associated with a lower risk of musculoskeletal disorders or whether such associations are comparable to those observed with the RA. For example, previous studies in other health domains have reported that the WW pattern may be associated with reductions in depression risk comparable to those observed with the RA.

However, a cross-sectional study conducted in a Korean population reported a more favorable association for the WW pattern than for the RA [22]. These inconsistencies may partly reflect differences in the operational definitions of the WW pattern across studies, as well as differences in the methods used to assess PA intensity, particularly between objective accelerometer-based measurements and subjective self-reported data. An additional research gap is that the existing literature combines evidence based on separate comparisons with inactive individuals with evidence from direct statistical comparisons between the WW and RA. Such methodological inconsistencies make it difficult to determine whether the WW pattern is associated with meaningful musculoskeletal health benefits and whether these associations are comparable to those observed with the RA. Moreover, it remains unclear whether concentrating a large proportion of weekly PA within a limited number of days may provide musculoskeletal benefits comparable to those associated with regularly distributed activity.

Accordingly, based on the available evidence, this review aims to address the following questions: (1) What is the association between the WW pattern and bone-related outcomes compared with IA, and how do these estimates compare with those for RA? (2) What is the association between the WW pattern and sarcopenia and other muscle-related outcomes compared with IA and RA? (3) Do these associations vary according to characteristics such as age, sex, body mass index, genetic susceptibility, and activity timing?

## 2. Methods

### 2.1. Search Strategy

This systematic review was undertaken in accordance with PRISMA 2000 quidelines. Three electronic databases, PubMed, Web of Science, and Scopus, were searched from their respective inception dates to 30 June 2026. The searches were restricted to English-language publications. No controlled vocabulary terms, such as Medical Subject Headings (MeSH), were used. Instead, the search was conducted using combinations of free-text keywords.

The search strategy combined the following terms: (“weekend warrior” OR “weekend warriors” OR “WW”) AND (“musculoskeletal system” OR “osteoporosis” OR “sarcopenia” OR “osteoarthritis” OR “bone mineral density” OR “BMD” OR “bone health” OR “musculoskeletal disorder”). In Scopus, the search was conducted using the title, abstract, and keyword fields (TITLE-ABS-KEY). The complete database-specific search strategies, including search fields and limits, are provided in Supplementary Table S2. To identify potentially eligible studies not retrieved by the electronic database searches, manual backward citation searching was performed by examining the reference lists of relevant studies. Specifically, Kim et al. [22] was identified through the reference list of S. Wang et al. [23]. This supplementary citation search was conducted on 1 July 2026.

The review was retrospectively registered with INPLASY on 1 September 2026, after its completion but before publication (registration number: INPLASY202690005). No substantive departures from the registered methods were identified.

Two investigators (C.H. and X.Z.) independently undertook literature screening according to the predefined eligibility criteria, data extraction using a standardized framework, and methodological appraisal using the designated assessment tools. Differences in judgment were discussed between the reviewers, with a third investigator (Q.L.) involved when agreement could not initially be reached.

### 2.2. Eligibility Criteria

#### 2.2.1. Inclusion Criteria

Studies were considered eligible when they satisfied all of the following conditions: (1) participants were adults aged 18 years or older; (2) the principal exposure represented a WW pattern defined according to the concentration of weekly PA within 1–2 days/sessions; (3) at least one relevant comparison group representing a lower level of PA, such as IA or insufficiently active, or RA participants, was included; (4) at least one musculoskeletal health outcome was reported; and (5) PA exposure was measured using either objective or self-reported methods.

#### 2.2.2. Exclusion Criteria

Studies were excluded when: (1) WW could not be distinguished from other PA patterns; (2) studies in which the WW pattern was not the main exposure of interest; (3) publication was a review, conference proceeding, or animal study; and (4) studies involving participants with musculoskeletal disorders but reporting outcome variables unrelated to musculoskeletal health.

### 2.3. Data Extraction

A standardized extraction framework was used to record information from each eligible study. The extracted variables included: (1) publication details and country; (2) source of the study population; (3) method of PA assessment; (4) sample size and participant characteristics; (5) study design and follow-up duration where applicable; (6) operational criteria used to classify WW, RA, and IA; (7) criteria used to define the outcome variables; (8) main findings and corresponding effect estimates; and (9) statistical methods used for the analyses.

Primary extraction was independently completed by two authors (C.H. and X.Z.), while the third (Q.L.) and fourth (Y.Y.) authors reviewed the extracted data for accuracy and completeness. The extracted data were also randomly cross-checked to minimize the potential for data extraction errors. Any inconsistencies identified during this process were resolved through discussion among the authors.

### 2.4. Data Synthesis

Data extraction included adjusted effect estimates, including hazard ratios (HRs), odds ratios (ORs), and β coefficients from linear regression models, together with their corresponding 95% confidence interval (CI). First, several studies used overlapping participant samples from large databases, such as the UK Biobank and the National Health and Nutrition Examination Survey (NHANES), which could introduce a risk of double-counting participants and biasing pooled estimates. Second, the operational definitions and measurement criteria for the WW pattern varied across studies, with different thresholds used to define WW and PA assessed using either objective accelerometer-based measures or subjective self-reported data, further increasing conceptual and methodological heterogeneity. Third, substantial methodological heterogeneity existed across studies in PA assessment methods, particularly between accelerometer-based measurements and questionnaire-based self-reports, as well as in the diagnostic criteria used for musculoskeletal conditions, such as sarcopenia. Given these sources of heterogeneity and the potential overlap of study populations, a quantitative meta-analysis was considered inappropriate, and the findings were therefore synthesized using a structured narrative synthesis supported by a structured data matrix. This approach enabled the direction and magnitude of the reported associations to be systematically compared across studies while also examining potential differences according to subgroup characteristics, such as age and sex. For each outcome, the most fully adjusted estimates from the primary analyses were prioritized, with direct WW–RA comparisons considered where available. The interpretation also considered the precision of estimates based on their 95% CIs, methodological quality, potential sources of bias and the directness of comparisons.

### 2.5. Methodological Quality Assessment

Different appraisal instruments were applied according to study design. Prospective cohort studies were evaluated using the Newcastle–Ottawa Scale (NOS) (available from: http://www.ohri.ca/programs/clinical_epidemiology/oxford.asp, accessed on 10 July 2026), which assesses three broad domains: participant selection, comparability between study groups, and outcome assessment. These domains consider aspects such as cohort representativeness, ascertainment of exposure, control for potential confounding, outcome assessment, and adequacy of follow-up. One star was awarded for each criterion met in the selection and outcome domains, with up to two stars for comparability. Unmet or unclear criteria received no stars. The maximum scores for the three domains were 4, 2, and 3 stars, respectively. Scores could range from 0 to 9 stars; studies receiving 0–3, 4–6, and 7–9 stars were categorized as low, moderate, and high quality, respectively. Cross-sectional investigations were assessed with the 11-item Agency for Healthcare Research and Quality (AHRQ) checklist. The checklist evaluates methodological aspects including the definition and selection of the study population, data sources and measurement procedures, quality assurance, handling of exclusions and missing data, control of confounding, and completeness of reporting. Each affirmative item was assigned one point, while negative or unclear responses received zero points. Total scores of 0–3, 4–7, and 8–11 were interpreted as indicating low, moderate, and high methodological quality. Detailed assessment criteria are provided in Supplementary Table S3.

Two reviewers (C.H. and X.Z.) independently completed all appraisals, and disagreements were settled through discussion or consultation with a third reviewer (Q.L.) until consensus was reached.

## 3. Results

### 3.1. Study Selection

**Figure 1:**
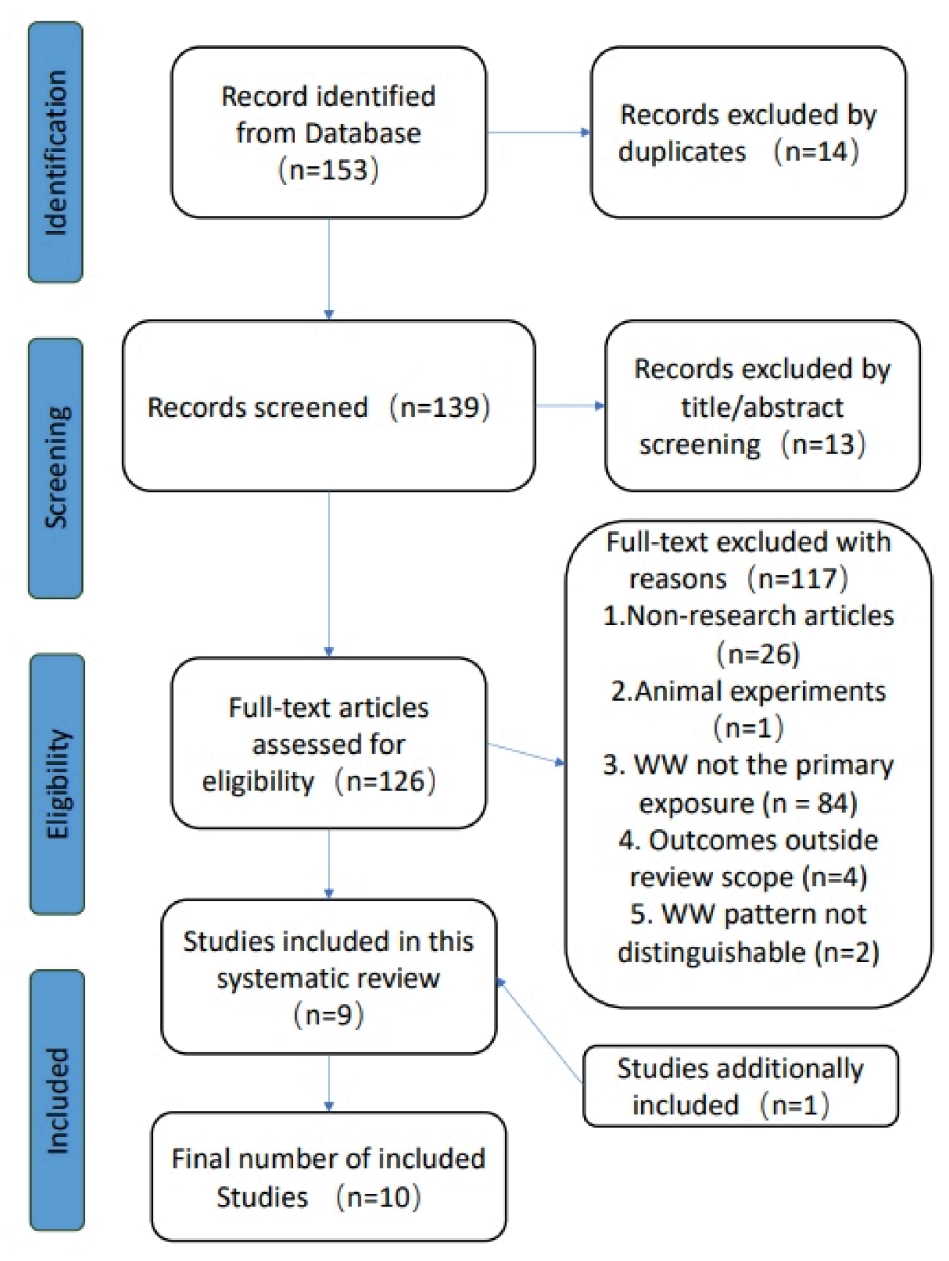
Flowchart showing the study selection process for the systematic review.

Figure 1 presents a flowchart summarizing the study selection process and the final inclusion of studies. The initial search yielded 153 potentially eligible records from databases. All retrieved records were consolidated into a single Microsoft Excel worksheet. Duplicate records were manually identified by comparing article titles, authors, publication years, and other bibliographic information. A total of 14 duplicate records were removed before title and abstract screening. After removing duplicates, screening titles and abstracts, and excluding ineligible articles, nine studies were identified through database searching. One additional study [22] was identified through supplementary backward citation searching. Thus, 10 studies [22,23,24,25,26,27,28,29,30,31] were ultimately included in the systematic review.

### 3.2. Characteristics of Included Studies

Table 1 summarizes the basic characteristics of the included studies. A total of 10 human studies were included in the analysis, comprising four cohort studies [24,27,28,31] and six cross-sectional studies [22,23,25,26,29,30]. Sample sizes varied across studies, ranging from 3539 to 83,376 participants. The data were derived from several large population-based databases: four studies [25,26,29,30] used data from the US NHANES, four [24,27,28,31] were based on the UK Biobank, one [23] used data from the China Health and Retirement Longitudinal Study (CHARLS), and one [22] used data from the Korea National Health and Nutrition Examination Survey (KNHANES). Participants in all included studies were aged 18 years or above, although the majority of studies primarily involved middle-aged and older adults. Overall, the included studies showed a relatively balanced distribution of male and female participants. PA was primarily assessed using accelerometer-based measurements or self-reported data. All included studies were published between 2025 and 2026. Specifically, five studies [25,26,28,29,30] were published in 2025 and five [22,23,24,27,31] in 2026. Among the cohort studies, follow-up durations ranged from 7.7 to 8.2 years. The included studies investigated the relationship between the WW pattern and multiple musculoskeletal outcomes, encompassing low bone mineral density, osteoporosis, osteoarthritis, degenerative spinal conditions, and sarcopenia.

Table 1 provides detailed information on participant demographics, study characteristics, and major covariates, whereas Table 2 summarizes the classification of different PA patterns and the methods used to assess PA. Table 3 presents the main outcome variables, their assessment criteria and measurement methods, and the corresponding effect estimates, while Supplementary Table S1 material provides a detailed summary of the operational definitions of the WW pattern used in each study because these classification criteria varied across studies.

### 3.3. Quality Assessment

The methodological quality assessments of the cohort and cross-sectional studies are presented in Table 4 and Table 5, respectively. The AHRQ scores of the six cross-sectional studies indicated generally good methodological quality. Most studies clearly defined the study population and assessment methods, although their main potential limitation was the inherent inability of cross-sectional designs to establish temporal or causal relationships. The NOS assessments likewise classified all four cohort studies as high quality. The main strengths of these studies included clearly defined participant selection criteria and good comparability between groups. Overall, the included studies were considered to be of generally high methodological quality.

### 3.4. Bone-Related Outcomes

#### 3.4.1. Bone Mineral Density

Three cross-sectional studies [25,29,30] evaluated the association between the WW pattern and bone mineral density (BMD), all three of which assessed PA using self-reported measures and quantified BMD via dual-energy X-ray absorptiometry, although the outcome definitions and statistical approaches differed between studies (Table 3). Y. Wang et al. [29] defined low BMD as a binary outcome and separately assessed low BMD at the total femur, femoral neck, femoral trochanter, intertrochanteric region, and lumbar spine, whereas H. Li & Wang [25] and Xu et al. [30] analyzed femoral neck BMD as a continuous outcome. H. Li & Wang [25] and Y. Wang et al. [29] performed direct statistical comparisons between WW and RA, whereas Xu et al. [30] did not.

Y. Wang et al. [29] reported that, compared with IA, WW and RA were associated with 35% (OR = 0.65, 95% CI: 0.46–0.92) and 24% (OR = 0.76, 95% CI: 0.62–0.93) lower odds of low BMD, respectively, with no statistically significant difference between WW and RA. Similarly, H. Li & Wang [25] reported higher femoral neck BMD among participants classified as WW or RA than among those classified as IA (WW: β = 0.034, 95% CI: 0.025–0.044; RA: β = 0.027, 95% CI: 0.020–0.033), whereas the difference in BMD between WW and RA was not statistically significant. Xu et al. [30] further found that, compared with IA, WW was associated with higher BMD at the total femur (β = 0.049, 95% CI: 0.007–0.090), femoral neck (β = 0.050, 95% CI: 0.010–0.097), trochanter (β = 0.041, 95% CI: 0.003–0.079), and intertrochanteric region (β = 0.048, 95% CI: 0.001–0.094), whereas RA showed no statistically significant associations at these sites. However, no direct comparison between WW and RA was reported in this study.

In further analyses, Y. Wang et al. [29] found that, compared with IA, WW was associated with lower odds of low BMD at the total femur (OR = 0.45, 95% CI: 0.26–0.75), femoral neck (OR = 0.50, 95% CI: 0.29–0.85), femoral trochanter (OR = 0.62, 95% CI: 0.39–0.99), and intertrochanteric region (OR = 0.33, 95% CI: 0.18–0.60), whereas the association with low lumbar spine BMD was not statistically significant (OR = 0.79, 95% CI: 0.53–1.18). In contrast, RA was significantly associated with lower odds of low BMD across all of the aforementioned skeletal sites.

Further subgroup and interaction analyses revealed variations in these associations according to BMI, sex, age, and marital status. BMI-stratified analyses showed that WW was associated with lower odds of low BMD among participants with BMI < 25 kg/m^2^ (OR = 0.63, 95% CI: 0.41–0.99), whereas the association did not reach statistical significance among those with BMI ≥ 25 kg/m^2^ (OR = 0.57, 95% CI: 0.31–1.02). RA also showed a statistically significant association only among participants with BMI < 25 kg/m^2^.

H. Li & Wang [25] and Y. Wang et al. [29] reported sex-stratified results, which suggested that the association between WW and BMD outcomes may be more pronounced in men than in women [25,29]. In the study by Y. Wang et al. [29], WW was associated with lower odds of low BMD in men (OR = 0.59, 95% CI: 0.39–0.89), whereas no statistically significant association was observed in women (OR = 0.87, 95% CI: 0.41–1.87). RA was likewise significantly associated with lower odds of low BMD only in men (OR = 0.71, 95% CI: 0.55–0.93). Similarly, H. Li & Wang [25] reported larger association estimates for femoral neck BMD with both WW and RA in men than in women. Xu et al. [30] also conducted subgroup analyses but found no statistically significant interactions by sex, age, race/ethnicity, educational attainment, or BMI.

Notably, H. Li & Wang [25] also identified statistically significant interactions by age and marital status (*p* < 0.01). Among participants aged ≤50 and >50 years, the association estimates for femoral neck BMD were β = 0.039 (95% CI: 0.026–0.053) and β = 0.034 (95% CI: 0.019–0.049) for WW, respectively, and β = 0.042 (95% CI: 0.032–0.053) and β = 0.013 (95% CI: 0.005–0.022) for RA, respectively. After stratification by marital status, the largest association estimates were observed among participants who had never married.

#### 3.4.2. Osteoporosis

Four studies [24,28,30,31] evaluated the associations of WW and RA with osteoporosis risk, including three cohort studies [24,28,31] and one cross-sectional study [30]. One study assessed PA exposure using self-reported measures, Xu et al. [30], while the remaining three utilized objective accelerometer-based measurements [24,28,31]. All four studies showed that, compared with IA, WW was associated with a lower risk of osteoporosis, while RA showed associations in the same direction. Huang et al. [24] and Zhu et al. [31] performed direct comparisons between WW and RA, whereas Song et al. [28] and Xu et al. [30] compared each activity pattern separately with IA as the common reference. The point estimates reported by Xu et al. [30] suggested a greater reduction in the odds of osteoporosis for WW than for RA relative to IA. However, because WW and RA were not directly compared, these findings cannot be interpreted as evidence that WW was superior to RA.

Zhu et al. [31] further used a multistate model to examine the associations of WW and RA with transitions between musculoskeletal disease states. For the transition from no musculoskeletal disease to osteoporosis, both WW (HR = 0.85, 95% CI: 0.73–0.98) and RA (HR = 0.78, 95% CI: 0.70–0.85) were associated with a lower risk of developing osteoporosis. However, for the subsequent transition from osteoporosis to musculoskeletal multimorbidity (MSM), neither WW (HR = 0.92, 95% CI: 0.63–1.34) nor RA (HR = 0.79, 95% CI: 0.50–1.25) showed a statistically significant association.

Furthermore, subgroup and interaction analyses provided additional information on potential variations in these associations according to genetic susceptibility, sex, age, and the timing of MVPA accumulation. After stratification by genetic risk, Huang et al. [24] found no statistically significant evidence that genetic susceptibility modified the association between WW and osteoporosis risk. In contrast, Song et al. [28] observed a statistically significant association between WW and lower osteoporosis risk only among participants at high genetic risk, whereas the associations did not reach statistical significance in the other genetic risk groups.

Sex-stratified analyses showed no statistically significant modification of the association between WW and osteoporosis risk by sex in Huang et al. [24], consistent with the absence of a significant sex interaction for osteoporosis reported by Song et al. [28]. Age-stratified analyses showed that, among participants aged ≥60 years, both RA (HR = 0.72, 95% CI: 0.63–0.83) and WW (HR = 0.72, 95% CI: 0.62–0.83) were associated with a lower risk of osteoporosis, whereas neither activity pattern showed a statistically significant association among participants aged <60 years. However, in the cross-sectional study by Xu et al. [30], WW was also associated with lower odds of osteoporosis among participants aged >50 years.

Song et al. [28] further classified participants into seven groups according to the predominant time window of MVPA accumulation: 03:00–06:00, 06:00–09:00, 09:00–12:00, 12:00–15:00, 15:00–18:00, 18:00–21:00, and 21:00–03:00. Because the early-afternoon group had the highest incidence density of osteoporosis, this group was used as the reference. Compared with the early-afternoon group, both the evening group (HR = 0.67, 95% CI: 0.52–0.87) and the morning group (HR = 0.69, 95% CI: 0.54–0.87) were associated with a lower risk of osteoporosis; the early-morning group also showed a statistically significant inverse association, although the magnitude was smaller (HR = 0.77, 95% CI: 0.64–0.92).

Beyond osteoporosis, Huang et al. [24] also examined incident fractures. Compared with IA, those following the WW pattern had a lower risk of fractures (HR = 0.77, 95% CI: 0.63–0.95). In a direct comparison with RA, WW also had a lower risk of fractures (HR = 0.72, 95% CI: 0.57–0.91).

#### 3.4.3. Other Outcomes

Among the 10 included studies, one cohort study [31] using accelerometer-based objective measurements of PA simultaneously evaluated several degenerative musculoskeletal outcomes, including osteoarthritis, spinal degenerative changes, and inflammatory arthritis. Using IA as the reference, the study separately estimated the associations of WW and RA with each outcome and further performed direct comparisons between WW and RA.

Compared with IA, WW was associated with a lower risk of osteoarthritis (HR = 0.83, 95% CI: 0.77–0.89), spinal degenerative changes (HR = 0.85, 95% CI: 0.78–0.94), and inflammatory arthritis (HR = 0.75, 95% CI: 0.58–0.98). RA was likewise associated with a lower risk of osteoarthritis (HR = 0.76, 95% CI: 0.69–0.83) and spinal degenerative changes (HR = 0.85, 95% CI: 0.76–0.95), whereas no statistically significant association was observed for inflammatory arthritis [31].

The study further applied a multistate model to examine the associations between different PA patterns and transitions across musculoskeletal disease states [31]. For the transition from no musculoskeletal disease to the first musculoskeletal condition, WW was associated with a lower risk of developing osteoarthritis (HR = 0.80, 95% CI: 0.74–0.88) and spinal degenerative changes (HR = 0.82, 95% CI: 0.77–0.87), while RA was also associated with lower risks of osteoarthritis (HR = 0.76, 95% CI: 0.70–0.82) and spinal degenerative changes (HR = 0.77, 95% CI: 0.70–0.86).

For the transition from a single musculoskeletal condition to musculoskeletal multimorbidity (MSM), both WW (HR = 0.71, 95% CI: 0.57–0.90) and RA (HR = 0.69, 95% CI: 0.52–0.93) were associated with a lower subsequent risk of MSM among participants who first developed osteoarthritis. Among participants who first developed spinal degenerative changes, WW was associated with a lower risk of progression to MSM (HR = 0.73, 95% CI: 0.56–0.96), whereas the association for RA did not reach statistical significance [31]. Direct comparisons between WW and RA showed no statistically significant difference in the risk of progression to MSM.

Sensitivity analyses using five alternative operational definitions of WW yielded broadly consistent results, with similar directions and magnitudes of association for osteoarthritis, spinal degenerative changes, and the corresponding disease-state transitions [31].

In addition, Zhu et al. [31] also examined gout and musculotendinous and soft tissue disorders (Supplementary Table S4). RA was associated with a lower risk of gout compared with inactivity, whereas the corresponding association for WW was not statistically significant. However, in a direct comparison, WW was associated with a higher risk of gout than RA (HR = 1.47, 95% CI: 1.11–1.94). Neither activity pattern showed a statistically significant association with musculotendinous and soft tissue disorders compared with inactivity, and no significant difference was observed between WW and RA (HR = 1.04, 95% CI: 0.95–1.14).

### 3.5. Sarcopenia

Out of the 10 articles included, four [22,23,26,27] evaluated the association between WW and sarcopenia, including three cross-sectional studies [22,23,26] and one cohort study [27]. PA was assessed using accelerometry in one study [27] and self-reported measures in other three studies [22,23,26], while the definitions and assessment methods for sarcopenia also varied across studies. Kim et al. [22] and Z. Li et al. [26] used anthropometrically estimated and DXA-derived muscle mass indices, respectively, without incorporating muscle strength or physical performance. Liu et al. [27] separately assessed probable and confirmed sarcopenia, whereas S. Wang et al. [23] combined possible and confirmed sarcopenia in its primary analysis (Table 3). All four studies showed that, compared with IA, WW was associated with a lower risk or lower odds of sarcopenia, and the magnitude of the associations was generally similar between WW and RA in most studies. Liu et al. [27] and Kim et al. [22] performed direct comparisons between WW and RA, whereas Z. Li et al. [26] and S. Wang et al. [23] used IA as the common reference and did not directly test differences between WW and RA.

In the only cohort study, Liu et al. [27] conducted sensitivity analyses using several operational definitions of WW and activity thresholds, including meeting the guideline-recommended weekly MVPA volume with ≥50% of MVPA accumulated within 1–2 days, ≥75% accumulated within 1–2 days, ≥50% accumulated within 1–2 consecutive days, and ≥50% accumulated within 1–2 weekend days. Across these alternative definitions, the association between WW and a lower risk of probable sarcopenia or sarcopenia was generally consistent in direction. When WW was defined as ≥150 min of MVPA per week with ≥50% accumulated within 1–2 days, WW remained significantly associated with a lower risk of probable sarcopenia, whereas the association for RA did not reach statistical significance; under the remaining definitions, both WW and RA were associated with a lower risk of (probable) sarcopenia, with broadly similar magnitudes of association. Furthermore, across all alternative WW definitions, both WW and RA were associated with lower risks of falls compared with IA, with no statistically significant differences observed between WW and RA.

S. Wang et al. [23] similarly examined the robustness of the findings using different weekly MVPA thresholds. When the MVPA threshold was set at ≥100 min/week, both WW (OR = 0.62, 95% CI: 0.42–0.94) and RA (OR = 0.63, 95% CI: 0.54–0.74) were associated with lower odds of the combined possible and confirmed sarcopenia outcome compared with IA. These associations remained evident when the threshold was increased to ≥125 min/week. However, when the MVPA threshold was further increased to ≥175 and ≥200 min/week, the associations with this combined sarcopenia outcome no longer reached statistical significance, with ORs of 0.75 (95% CI: 0.47–1.20) and 0.74 (95% CI: 0.46–1.18), respectively. Notably, the analysis of confirmed sarcopenia yielded an imprecise estimate (OR = 0.99, 95% CI: 0.44–2.22), leaving the association between WW and confirmed sarcopenia uncertain.

Further analyses suggested that the associations between PA patterns and sarcopenia varied across several demographic and behavioral subgroups. Subgroup analyses by Liu et al. [27] showed a statistically significant interaction between age and PA pattern. Among participants aged <65 years, the inverse associations of WW and RA with probable sarcopenia or sarcopenia were more pronounced, whereas similar associations were not observed among those aged ≥65 years. Sex-stratified analyses suggested that the inverse associations of WW and RA with sarcopenia were more pronounced in women, in contrast to the findings of S. Wang et al. [23], in which the association for WW appeared stronger in men.

Z. Li et al. [26] further examined the associations of sedentary time, total PA duration, and activity intensity with sarcopenia. Each additional hour of daily sedentary time was associated with 5% higher odds of sarcopenia (OR = 1.05, 95% CI: 1.01–1.10), with a linear dose–response relationship, whereas each additional hour of daily PA was associated with 6% lower odds of sarcopenia, also following a linear relationship. Then analyzed by activity intensity, VPA showed a relatively strong inverse association with sarcopenia (OR = 0.39, 95% CI: 0.29–0.53). Age- and sex-stratified analyses showed relatively larger effect estimates among participants aged 45–59 years and among men.

Subgroup analyses by S. Wang et al. [23] showed that, compared with IA, WW was significantly associated with lower odds of sarcopenia among married participants, rural residents, and those sleeping 6–8 h per night. RA was associated with lower odds of sarcopenia across most subgroups, although the associations did not reach statistical significance among participants with a high-school education or above, those sleeping >9 h per night, and those who were underweight.

However, in a direct comparison, Kim et al. [22] found that participants in WW had 75% lower odds of sarcopenia than those in RA. In further analyses of activity intensity, after adjustment for PA frequency, higher VPA remained associated with lower odds of sarcopenia (OR = 0.54, 95% CI: 0.33–0.86), whereas the association between higher MPA and sarcopenia was no longer statistically significant (OR = 0.87, 95% CI: 0.66–1.15).

## 4. Discussion

This systematic review comprehensively synthesized the available evidence on the associations between the WW pattern and musculoskeletal disorders. Overall, the findings indicated that, compared with IA, accumulating the recommended weekly volume of MVPA within 1–2 days was generally associated with a lower risk or lower odds of low bone mineral density, osteoporosis, osteoarthritis, spinal degenerative changes, inflammatory arthritis, and sarcopenia. Despite some variation across subgroups and study designs, the available evidence generally suggests that, for most outcomes, the direction and magnitude of the associations observed for WW were broadly comparable to those observed for RA. These findings suggest that, for musculoskeletal health, achieving an adequate total volume of weekly PA may be associated with favorable outcomes under both concentrated and regularly distributed activity patterns. However, this interpretation should be made cautiously because the current evidence is derived predominantly from observational studies, definitions of WW and musculoskeletal outcomes were not fully consistent across studies, and some subgroup findings showed considerable heterogeneity.

The evidence regarding bone health was generally consistent, although the findings were not uniform across skeletal sites or population subgroups. Studies that directly compared PA patterns generally showed that WW was associated with lower odds of low BMD compared with IA, whereas no clear differences were identified between WW and RA [25,29]. Thus, provided that the recommended total volume of PA is achieved, concentrating activity within fewer days does not appear to be associated with an overall disadvantage in BMD. However, in one study, WW was not associated with significantly lower odds of low BMD at the lumbar spine. In contrast, RA showed significant inverse associations with low BMD across all skeletal sites assessed, including the total femur, femoral neck, trochanteric and intertrochanteric regions, and lumbar spine [29]. This site-specific finding may reflect differences in mechanical loading, measurement precision, or statistical power, and also suggests that the association between WW and bone health should not be assumed to be uniform across all skeletal sites.

At the same time, the relationship between WW and low BMD also appeared to differ across BMI and sex subgroups, although these findings remain exploratory. One analysis showed that WW was associated with lower odds of low BMD among participants with a BMI < 25 kg/m^2^, but no statistically significant association was observed in those with a BMI ≥ 25 kg/m^2^. A comparable pattern was reported for RA [29]. The greater mechanical loading associated with higher body weight, together with the potentially lower risk of low BMD observed in some individuals with higher BMI [32,33], may attenuate detectable differences between physically active and inactive groups. However, a statistically significant association in one subgroup and a non-significant association in another do not necessarily indicate a true difference in effect between the subgroups. Confirmation of effect modification therefore requires formal interaction testing and adequately powered subgroup analyses.

Similarly, the relatively stronger associations observed in men may be related to differences in testosterone levels [34,35] and exercise intensity [36,37,38]. Sex hormones may contribute to an anabolic environment that supports bone remodeling [39,40,41], while higher-intensity mechanical loading may activate osteocytic signaling pathways [42] and stimulate the release of osteogenic mediators, including myokines, lactate, and anabolic hormones [43,44,45]. However, these mechanisms were not directly examined in the included studies. Accordingly, sex hormones should be considered a biologically plausible explanation for the observed associations rather than a confirmed causal pathway.

Compared with IA, WW was consistently associated with a lower risk of osteoporosis, with effect estimates generally comparable to those observed for RA [24,28,30]. However, the available evidence remains inconsistent regarding whether genetic susceptibility and age modify this association.

Huang et al. [24] found no clear differences in the association across genetic risk levels, whereas Song et al. [28] observed a statistically significant association only among individuals at high genetic risk. Differences in sample characteristics, outcome ascertainment, statistical power, and methods used to stratify genetic risk may have contributed to these inconsistent findings. Moreover, age-stratified findings showed some heterogeneity. One study [24] reported inverse associations of both WW and RA with osteoporosis risk in adults aged ≥60 years, while no statistically significant associations were observed among participants younger than 60 years. Notably, the incidence of osteoporosis in the younger subgroup was only 1.2%, and the limited number of outcome events may have reduced statistical power and increased uncertainty in the effect estimates. The absence of statistical significance in this subgroup should not be taken to indicate that WW is unrelated to osteoporosis risk in adults younger than 60 years.

Beyond the PA pattern itself, the timing of it may represent another factor of potential relevance. This provides a potentially important preliminary perspective for understanding the association between WW and osteoporosis risk. Song et al. [28] examined activity timing among participants accumulating at least 150 min of MVPA per week and found that, compared with activity performed in the early afternoon, activity accumulated in the morning and evening was associated with a lower risk of osteoporosis (morning: HR = 0.69, 95% CI: 0.54–0.87; evening: HR = 0.67, 95% CI: 0.52–0.87), while early-morning activity showed a weaker but still statistically significant association (HR = 0.77, 95% CI: 0.64–0.92), suggesting that activity timing may be related to circadian rhythms in bone metabolism. However, these analyses were not restricted to WW participants and therefore do not establish a preferred exercise time for this group. Previous physiological evidence indicates that osteocyte energy metabolism, sex hormone levels, core body temperature, and joint mobility exhibit circadian variation. These factors could theoretically influence the skeletal response to mechanical loading. For example, morning elevations in testosterone may further promote the differentiation and proliferation of osteogenic cells [46,47]. Nevertheless, these mechanisms were not directly examined in the included studies, and comparisons across multiple time windows may increase the likelihood of chance findings. Therefore, before activity timing is incorporated into exercise recommendations, these findings require further confirmation using prespecified hypotheses, objective PA monitoring, and repeated-measures designs.

From a biological perspective, concentrated PA may influence bone health through repeated mechanical loading and metabolic stimulation. Higher-intensity mechanical loading may promote osteogenic adaptation, and previous studies have also suggested that resistance and impact-based exercise may improve BMD and modulate signaling pathways involved in bone formation [42,43,45]. Intermittent mechanical and metabolic stimuli may also influence growth hormone, insulin-like growth factor 1, interleukin-6, adiponectin, and signaling pathways involved in osteoblast proliferation and differentiation [36,38,48]. These findings are consistent with the interpretation that mechanical stimuli of sufficient intensity may be beneficial for bone health [37,49,50]. Importantly, however, WW describes the temporal distribution of weekly PA and should not be equated with high-intensity interval training or impact-based exercise. Because most included studies did not distinguish exercise modalities in detail, the current evidence cannot determine whether the observed associations are attributable to the concentrated distribution of activity, a greater proportion of VPA, specific mechanical loading characteristics, or differences in total energy expenditure. Xu et al. [30] also observed that, compared with the sedentary group, both WW and RA were associated with a lower risk of osteoporosis, with a larger effect estimate observed for WW [30]. This difference in effect estimates may reflect differences in activity patterns; however, the possibility that a higher weekly exercise frequency could contribute to insufficient skeletal recovery, accelerated bone resorption, or increased oxidative stress remains speculative and has not been directly established in the included studies [51].

Notably, the potential benefits associated with WW may extend beyond BMD and osteoporosis. Compared with IA, WW was also associated with a lower risk of osteoarthritis, spinal degenerative changes, and inflammatory arthritis, with effect estimates generally comparable to those observed for RA [31]. The direction and magnitude of these associations remained broadly consistent across five alternative definitions of the PA patterns, supporting the robustness of the findings. Disease-state transition analyses further suggested that WW was associated not only with a lower risk of developing an initial musculoskeletal condition, but also with a lower risk of progression from a single condition to MSM, defined as the coexistence of at least two of osteoarthritis, spinal degenerative changes, and osteoporosis. Among participants who first developed osteoarthritis, both WW and RA were associated with a lower subsequent risk of MSM, whereas among those who first developed spinal degenerative changes, only the association for WW reached statistical significance [31]. However, a direct comparison showed that WW was associated with a modestly higher hazard of osteoarthritis than RA (HR = 1.11, 95% CI: 1.02–1.20). These findings suggest that concentrated PA may be relevant at different stages of musculoskeletal disease progression; however, they do not establish that WW can prevent or halt the development of musculoskeletal multimorbidity. Because early symptoms or functional limitations may themselves influence subsequent PA patterns, the possibility of reverse causation cannot be excluded.

Regarding sarcopenia, although the diagnostic criteria varied across studies, all included studies showed that WW was associated with a lower risk or lower odds of sarcopenia compared with IA, with the magnitude of the association generally comparable to that observed for RA [23,26,27]. However, the evidence regarding confirmed sarcopenia remains inconclusive, particularly given the imprecise estimate reported by S. Wang et al. [23].

Across alternative definitions and thresholds for WW, the direction of the association with sarcopenia was generally consistent; however, when WW was defined as meeting the guideline-based threshold of ≥150 min of MVPA per week with ≥50% of total MVPA accumulated within 1–2 days, RA was not significantly associated with probable sarcopenia. This finding was not entirely consistent with that of S. Wang et al. [23], in which the association between WW and sarcopenia no longer reached statistical significance when more stringent MVPA thresholds of 175 and 200 min/week were applied (OR = 0.75, 95% CI: 0.47–1.20; OR = 0.74, 95% CI: 0.46–1.18, respectively).

Findings stratified by age and sex were also inconsistent, with Liu et al. [27] reporting stronger inverse associations between PA patterns and probable sarcopenia or sarcopenia among participants younger than 65 years, but not among those aged 65 years or older. Liu et al. [27] also reported more pronounced inverse associations of WW and RA with probable sarcopenia and sarcopenia among women, whereas Z. Li et al. [26] observed relatively larger association estimates among participants aged 45–59 years and among men. S. Wang et al. [23] similarly reported a significant association between WW and lower odds of sarcopenia in men. One possible biological explanation for these sex differences may involve sex hormone levels, as declining estrogen levels in women may contribute to reductions in muscle mass and strength and thereby attenuate potential benefits for sarcopenia-related outcomes [52]. Overall, the current evidence regarding age and sex-specific differences remains inconclusive.

Activity intensity may partly explain why concentrated and regularly distributed activity patterns show broadly similar overall associations with sarcopenia. In the direct comparison conducted by Kim et al. [22], WW was associated with 75% lower odds of sarcopenia defined using anthropometrically estimated muscle mass than RA; participants classified as WW accumulated less moderate-intensity activity but more VPA than those classified as RA, despite similar total weekly energy expenditure between the two groups. Together with the stronger inverse association between VPA and sarcopenia reported by Z. Li et al. [26], these findings suggest that activity intensity may provide additional explanatory information beyond total activity volume and frequency. From a mechanistic perspective, aerobic exercise may enhance mitochondrial ATP production in skeletal muscle and promote muscle protein synthesis [53], whereas resistance exercise may promote muscle hypertrophy and strength by regulating the balance between protein synthesis and degradation [54]. However, extremely concentrated or excessive PA may not confer additional benefits, potentially because excessive exercise can contribute to muscle damage, impaired muscle growth, and greater physiological and psychological stress associated with high-intensity PA [26]. In addition, the adverse association between sedentary time and sarcopenia suggests that prolonged sedentary behavior, particularly when it displaces PA, may adversely affect muscle health by promoting chronic inflammation and fat accumulation [55], which may contribute to muscle atrophy and exacerbate anabolic resistance [56,57].

The practical interpretation of these findings should consider both feasibility and safety. For individuals who find it difficult to schedule exercise on most days of the week, WW may represent a more feasible alternative, particularly when the recommended total volume of weekly PA can be achieved. However, concentrating a large volume of PA into 1–2 sessions may increase acute mechanical loading, which warrants particular consideration for older adults, individuals with lower baseline physical fitness, and those with pre-existing musculoskeletal conditions. Although Hartnett et al. [58] discussed shoulder and elbow injuries among recreational weekend warriors in a narrative review, whether WW is associated with a higher incidence of musculoskeletal injury than RA remains uncertain. Beyond these safety considerations, RA may theoretically provide more sustained cardiovascular benefits, such as reductions in blood pressure and blood lipid levels, although these potential advantages over WW have not been directly established [20]. The available evidence neither supports considering RA an overall inferior option because of concerns about “overtraining” nor provides sufficient evidence to establish that WW is superior to RA. Therefore, when concentrated PA schedules are implemented in practice, emphasis should be placed on progressive increases in training load, appropriate selection of exercise modalities, and individualized risk assessment.

## 5. Strengths and Limitations

This review has several notable features. First, using the World Health Organization recommendations for weekly MVPA among adults as an important reference, this review systematically synthesized the available evidence on the associations between WW and a broad range of musculoskeletal outcomes. This systematic review provides one of the earliest comprehensive syntheses of the available evidence linking the WW pattern to musculoskeletal health and helps fill a notable gap in the existing literature. Second, this review not only examined the associations of WW relative to IA, but also systematically evaluated differences between WW and RA while considering alternative WW definitions, activity thresholds, skeletal sites, and subgroup characteristics such as age, sex, body mass index, and genetic risk. This comprehensive perspective helps clarify whether the temporal distribution of PA remains relevant to musculoskeletal health once the recommended weekly activity volume is achieved and may inform the development of more flexible and feasible public health recommendations.

However, the findings of this review should be interpreted in light of several limitations. First, all included evidence was derived from cohort or cross-sectional studies, with no randomized controlled trials directly comparing different temporal distributions of PA. Therefore, causal inferences cannot be made. The observed inverse associations may still be influenced by residual confounding, healthy participant bias, and reverse causation. For example, individuals who have already experienced loss of muscle mass, low BMD, joint pain, or functional limitations may reduce their overall activity levels and particularly avoid higher-intensity activity concentrated within a few days, potentially leading to an overestimation of the association between WW and lower disease risk. Other factors that were not adequately measured or adjusted for, including diet quality, calcium and vitamin D intake, menopausal status, socioeconomic status, previous exercise habits, and sedentary behavior, may have further influenced the effect estimates.

Measurement error in PA assessment also limits the certainty of the evidence. Most studies relied on self-reported activity frequency, duration, and intensity, which are susceptible to recall and social desirability biases, and reporting errors may directly affect the classification of WW when activity is concentrated within 1–2 days. Some large cohorts, such as the UK Biobank, used wrist-worn accelerometers to provide objective measurements and improve the reliability of PA estimates. However, these devices typically capture activity over a limited monitoring period and may not fully represent long-term activity patterns. In addition, wrist-worn devices have limited ability to distinguish specific exercise modalities, such as resistance, aerobic, and flexibility exercise, and may not adequately quantify external mechanical load. Therefore, high scores on conventional methodological quality assessment tools do not necessarily indicate high overall certainty of evidence.

Another important methodological limitation is the lack of a consistent operational definition of WW across studies, including variation in weekly MVPA thresholds and the proportion of activity required to be concentrated within a limited number of days. These differences may affect participant classification and effect estimates, thereby limiting direct comparisons across studies. More importantly, some studies used IA as a common reference and separately estimated the associations of WW versus IA and RA versus IA, subsequently inferring comparable benefits from the similarity of the two effect estimates. Such indirect comparisons based on a common reference group cannot substitute for formal statistical comparisons directly contrasting WW with RA.

In addition, the literature search was restricted to PubMed, Web of Science, and Scopus. These databases were selected because PubMed provides extensive coverage of the biomedical and health-related literature, while Web of Science and Scopus offer broad multidisciplinary indexing and citation coverage, thereby providing complementary access to the literature relevant to musculoskeletal health and PA. Although supplementary backward citation searching was performed, the exclusion of other potentially relevant databases may have limited the sensitivity and comprehensiveness of the search and could have resulted in some eligible studies being missed.

Moreover, subgroup analyses and the generalizability of the findings remain limited. Some analyses stratified by age, sex, BMI, genetic risk, and activity timing included relatively few outcome events and involved multiple comparisons, and statistical significance in one subgroup but not another does not by itself demonstrate a true interaction. Although several original studies conducted subgroup analyses, evidence remains insufficient for individuals with existing musculoskeletal disorders, low baseline physical fitness, or a high risk of exercise-related injury. The included populations covered a broad age range but predominantly consisted of middle-aged and older adults. Therefore, the extent to which these findings can be generalized to frail, severely osteoporotic, or mobility-limited populations remains uncertain.

Finally, most included studies enrolled participants across a broad age range, with middle-aged and older adults accounting for a substantial proportion of the samples. With advancing age, concentrating a large volume of PA within 1–2 days may raise concerns about acute mechanical loading, particularly when individuals have limited training experience, inadequate warm-up, or excessively rapid progression in exercise intensity. Although WW may be associated with favorable bone health outcomes, Whether WW carries a greater risk of musculoskeletal injury than RA remains uncertain, and these potential risks should therefore be carefully considered when recommending this activity pattern.

Despite the inclusion of high-quality epidemiological evidence, well-designed large-scale randomized controlled trials (RCTs) and multi-omics studies are still needed to confirm and extend the current findings. Future studies should use repeated objective PA monitoring and standardized definitions of WW, prioritize direct comparisons between WW and RA, and comprehensively document exercise modality, intensity, load progression, and previous training experience.

Given the potential feasibility constraints of conventional randomized trials using long-term musculoskeletal disease outcomes, large prospective cohorts, pragmatic randomized trials, and mechanistic studies could be combined to strengthen causal inference. For older adults and individuals with lower baseline physical fitness, exercise programs should emphasize gradual adaptation to increasing loads, adequate warm-up, appropriate technical instruction, and individualized risk assessment. Further clarification of the optimal exercise modalities, intensity progression, and injury-prevention strategies for WW schedules will be important for improving feasibility while maximizing their potential effectiveness and safety.

## 6. Implications

The findings of this systematic review suggest that WW is associated with favorable musculoskeletal health outcomes in adults and may represent a practical and time-efficient approach to PA. Given that the associations observed for WW were broadly comparable to those for RA, public health strategies should encourage adults to accumulate sufficient PA in ways that are compatible with their individual schedules. Concentrating an adequate weekly volume of activity within 1–2 days may therefore represent a feasible alternative for maintaining musculoskeletal health among individuals who are unable to engage in more regularly distributed activity because of time constraints. Nevertheless, appropriate warm-up, gradual progression of exercise intensity, and adequate recovery should be emphasized when adopting concentrated activity schedules to minimize the potential risk of musculoskeletal injury associated with high-intensity or high-volume activity performed over a limited number of days.

## 7. Conclusions

Available observational evidence indicates that adults who satisfy weekly MVPA recommendations while concentrating activity into 1–2 days generally have more favorable musculoskeletal outcomes than inactive adults. For most musculoskeletal outcomes examined, the magnitude of association reported for WW was broadly similar to that reported for RA. However, individual studies reported a higher hazard of osteoarthritis but a lower hazard of fractures with WW compared with RA. These findings should be interpreted cautiously and do not establish overall superiority or equivalence between the two activity patterns. A concentrated schedule may therefore represent a practical means of achieving recommended PA levels for adults who cannot exercise on several days each week. Although WW shows promise as a flexible PA pattern associated with a lower risk of musculoskeletal outcomes, differences in activity thresholds, including duration and intensity, as well as age, sex, BMI, genetic susceptibility, and activity timing may define the boundaries of the current evidence and its generalizability.

Future investigations should use standardized WW criteria, repeated objective assessments of PA, and study designs capable of directly comparing concentrated with regularly distributed activity. Detailed reporting of exercise modality and injury outcomes will also be necessary to determine which populations can adopt a WW approach effectively and safely.

## Figures and Tables

**Table 1:** Basic characteristics of included studies.

Author	Data Source	Study Type	Sample Size (%)	Median Follow-Up (Years)	Females (%)	Baseline Age (Years) Mean (SD) or Median (Q25%, Q75%)	Age Range (Years)	Covariates Adjusted for
Huang et al. [24]	UK Biobank	Prospective cohort	Total: 14,069 Inactive: 4704 Regular: 3241 WW: 6124	7.8	7680 (54.59)	56.2 (7.8)	40–69	Age, race, educational level, deprivation, smoking and alcohol use, dietary score, BMI, study site, and fasting duration.
Kim et al. [22]	Korea National Health and Nutrition Examination Survey (KNHANES)14–22	Cross-sectional study	Total: 28,918 Inactive: 26,540 Regular: 2058 WW: 320	NA	17,172 (59.4)	>20	>20	Age, sex, household income, medication use, alcohol and smoking behaviors, BMI, weekly sedentary time, and total energy intake.
H. Li & Wang [25]	NHANES 2007–2010, 2013–2014, 2017–2020	Cross-sectional study	Total: 16,937 Inactive: 9123 Insufficiently active: 2559 Regular: 4303 WW: 952	NA	8260 (48.8)	53.6 (17.2)	≥18	Age, sex, race, educational attainment, marital status, poverty-to-income ratio, BMI, alcohol use, serum cotinine, calcium, phosphorus, and total protein levels, diabetes, and dyslipidemia.
Liu et al. [27]	UK Biobank	Prospective cohort	Total: 83,376 Inactive: 27,866 Regular: 19,542 WW: 35,968 Probable sarcopenia: 20,984 Sarcopenia: 20,294 Falls: 83,376	7.8	47,239 (56.7)	56.2 (7.8)	40–69	Age, sex, ethnicity, assessment center, smoking and alcohol use, accelerometer wear season, BMI, Townsend deprivation index, shift-work status, employment status, and healthy diet score.
Z. Li et al. [26]	NHANES (2011–2018)	Cross-sectional study	Total: 7869 Inactive: 4718 Regular: 2573 WW: 578	NA	3915 (49.15)	40 (29.0, 50.0)	18–59	Age, sex, ethnicity, educational attainment, marital status, PIR, alcohol and smoking behaviors, sleep duration, diet quality, and self-reported hypertension, diabetes, and cardiovascular disease.
Song et al. [28]	UK Biobank	Prospective cohort	Total: 69,310 Inactive: 22,164 Regular: 25,348 WW: 21,798	7.7	37,506 (54.1)	61.7 (7.9)	40–69	Age, sex, ethnicity, employment status, university or college education, TDI, genetic risk category, serum vitamin D and calcium levels, obesity, sleep, sedentary behavior, smoking and alcohol use, falls during the previous year, fracture history, hypertension, depression, diabetes, chronic kidney and liver disease, self-rated health, and vitamin D and calcium supplementation.
S. Wang et al. [23]	China Health and Retirement Longitudinal Study (CHARLS)	Cross-sectional study	Total: 3579 Inactive: 1492 Insufficiently active: 175 Regular: 1781 WW: 131	NA	1774 (49.6)	≥60	≥60	Age, sex, marital status, educational level, residential area, employment status, smoking and alcohol use, nighttime sleep duration, BMI, and comorbidity count.
Y. Wang et al. [29]	NHANES 2007–2008, 2009–2010, 2013–2014, 2017–2020	Cross-sectional study	Total: 6482 Inactive: 3020 Insufficiently active: 1038 Regular: 2098 WW: 326	NA	3231 (49.85)	42.45 (11.7)	20–60	Age, sex, race, BMI, comorbidity status, alcohol use, and serum cotinine, calcium, and phosphorus levels.
Xu et al. [30]	NHANES 2017–2020	Cross-sectional study	Total: 3539 Inactive: 2547 Regular: 892 WW: 100	NA	1667 (47.1)	64.46 (9.0)	>50	Age, sex, race/ethnicity, educational level, PIR, sedentary duration, BMI, waist circumference, alcohol and tobacco use, weekday sleep duration, and weekend sleep duration.
Zhu et al. [31]	UK Biobank	Prospective cohort	Total: 52,606 Inactive: 15,949 Regular: 13,542 WW: 23,115	8.2	29,259 (55.6)	55.1 (7.9)	40–69	Age, sex, and race TDI, and BMI, smoking status, alcohol consumption, diabetes, hypertension, and cancer.

Note: BMI, body mass index; PIR poverty income ratio; TDI, Townsend Deprivation Index. For Zhu et al. [31], the primary analytical cohort included 52,606 participants, while the MSM staging and subtype-specific analyses included 65,982 and 65,556 participants, respectively. PA-pattern-specific sample sizes for these additional analyses were not separately reported in the original study.

**Table 2:** Weekend warrior assessment and methodological characteristics of the included studies.

Author	Country (First Author)	Main Physical Activity Assessment	Physical Activity Patterns	PA Measurement Methods	Main Analytical Methods
Huang et al. [24]	China	Accelerometry	Inactive/WW/Regularly active	Axivity AX3 wrist-mounted triaxial accelerometer	Elastic net regression; Cox proportional hazards regression
Kim et al. [22]	Korea	Self-reported	Inactive/WW/Regularly active	Global Physical Activity Questionnaire (GPAQ)	Multivariable logistic regression
H. Li & Wang [25]	China	Self-reported	Inactive/Insufficiently active/WW/Regularly active	Global Physical Activity Questionnaire (GPAQ)	Survey-weighted multivariable linear regression
Liu et al. [27]	China	Accelerometry	Inactive/WW/Regularly active	Axivity AX3 wrist-worn triaxial accelerometer	Multivariable Cox proportional hazards regression
Z. Li et al. [26]	China	Self-reported	Inactive/WW/Regularly active	Global Physical Activity Questionnaire (GPAQ)	Survey-weighted logistic regression
Song et al. [28]	China	Accelerometry	Inactive/WW/Regularly active	Axivity AX3 wrist-worn triaxial accelerometer	K-means clustering; Cox proportional hazards regression
S. Wang et al. [23]	China	Self-reported	Inactive/Insufficiently active/WW/Regularly active	International Physical Activity Questionnaire—Short Form (IPAQ-SF)	Multivariable logistic regression
Y. Wang et al. [29]	China	Self-reported	Inactive/Insufficiently active/WW (Sufficiently active)/Regularly active	Standardized NHANES PA questionnaire; interview-based assessment	Survey-weighted multivariable linear and logistic regression
Xu et al. [30]	China	Self-reported	Inactive/WW/Regularly active	Global Physical Activity Questionnaire (GPAQ)	Survey-weighted multivariable logistic and linear regression
Zhu et al. [31]	China	Accelerometry	Inactive/WW/Regularly active	Axivity AX3 wrist-worn triaxial accelerometer	Cox proportional hazards regression

**Table 3:** Main outcome definitions, measurement methods, and effect estimates across the 10 included studies.

**Author**	**Main Outcome(s)**	**Operational Definition/Criterion**	**Measurement/Ascertainment Method**	**Main Outcome Effect Estimate (Fully Adjusted)**
Huang et al. [24]	Osteoporosis	Incident osteoporosis and fracture were defined by ICD-10 diagnosis codes.	Outcomes were ascertained through the UK Biobank First Occurrences registry, which combines primary care, hospital inpatient, death-registry and baseline self-reported data.	The WW pattern was associated with a lower risk of osteoporosis, while no statistically significant difference in osteoporosis risk was observed between WW and regularly active participants. Inactive = reference. WW: osteoporosis HR 0.59 (0.44–0.79); RA: osteoporosis HR 0.75 (0.54–1.06); Direct WW vs. RA: osteoporosis HR 0.71 (0.50–1.01) WW: fracture HR: 0.77 (0.63–0.95); RA: fracture HR: 1.06 (0.85–1.33); Direct WW vs. RA: fracture HR 0.72 (0.57–0.91).
Kim et al. [22]	Sarcopenia	Sarcopenia index = eASM/body mass × 100. Sarcopenia was defined as an index 2 SD below the sex-specific mean of the young reference group (20 to <40 years): <29.1% in men and <23.7% in women.	Appendicular skeletal muscle mass was estimated, not directly scanned, using the Shi anthropometric equation: eASM = 0.485 × 0.998^^^age ^×^ 0.814^^^(female) ^×^ 1.006^^^height ^×^ body mass^^^0.680	Compared with inactive and RA individuals, those following the WW pattern showed a lower prevalence of sarcopenia, indicating that concentrating relatively high-intensity activity into fewer sessions may be associated with more favorable sarcopenia outcomes. Inactive = reference. WW: OR 0.16 (0.07–0.35); RA: OR 0.64 (0.52–0.79); Direct WW vs. RA: OR 0.25 (0.11–0.56).
H. Li & Wang [25]	BMD	Continuous femoral neck BMD (g/cm^2^); no disease threshold applied.	Hologic Discovery Model A DXA with Apex software version 3.2. The left hip was scanned routinely; the right hip was used if the left had a fracture, replacement or fixation device.	The WW pattern was associated with higher BMD, with an effect magnitude similar to that observed for the RA. Inactive = reference. WW: β 0.034 (0.025–0.044); RA: β 0.027 (0.020–0.033); Direct WW vs. RA: β 0.008 (−0.003 to 0.018), *p* = 0.151.
Liu et al. [27]	(Probable) Sarcopenia	EWGSOP2: probable sarcopenia = grip strength < 27 kg in men or <16 kg in women; confirmed sarcopenia = low grip strength plus low muscle mass.	Grip strength was measured with a Jamar J00105 hydraulic hand dynamometer.	Concentrating weekly PA within 1–2 days was associated with a sarcopenia risk comparable to that observed with the RA. Inactive = reference. RA/WW; Direct WW vs. RA: Probable sarcopenia: HR 0.90 (0.78–1.03)/0.79 (0.70–0.89); 0.87 (0.76–1.00) Sarcopenia: HR 0.80 (0.65–0.97)/0.74 (0.63–0.87); 0.93 (0.77–1.13); Falls: HR 0.83 (0.75–0.92)/0.79 (0.72–0.85)/0.94 (0.85–1.04); Recurrent falls: HR 0.86 (0.76–0.96)/0.82 (0.75–0.90)/0.95 (0.85–1.07).
Z. Li et al. [26]	Sarcopenia	FNIH definition based on ASM/BMI: <0.789 in men and <0.512 in women.	ASM was obtained from NHANES dual-energy X-ray absorptiometry (DXA) data and processed using Hologic APEX software.	Both WW and RA exercise patterns were associated with a lower risk of sarcopenia. Inactive = reference. WW: OR 0.41 (0.23–0.72); RA: OR 0.71 (0.54–0.92); Direct WW vs. RA: not reported.
Song et al. [28]	Osteoporosis	ICD-10 M80, M81 or M82 in the UK Biobank First Occurrence field.	Outcomes were ascertained through the UK Biobank First Occurrences registry, which combines primary care, hospital inpatient, death-registry and baseline self-reported data.	Concentrating most weekly MVPA within 1–2 days was associated with bone health benefits comparable to those observed with the RA. Inactive = reference. WW: HR 0.78 (0.69–0.89); RA: HR 0.77 (0.67–0.87); Direct WW vs. RA: not reported.
S. Wang et al. [23]	(Possible) Sarcopenia	AWGS 2019. Possible sarcopenia = low strength with or without low performance; confirmed sarcopenia = low muscle mass plus low strength or poor performance. Low strength: <28 kg men/<18 kg women. Low performance: five-chair-stand time ≥ 12 s or gait speed < 1.0 m/s. Low ASM/height^2^: <6.90 kg/m^2^ men/<5.08 kg/m^2^ women.	Grip: Yuejian WL-1000 dynamometer, two trials per hand, maximum dominant-hand value. Performance: five rises from a 47-cm armless chair and two usual-pace 2.5-m walks. ASM was estimated from age, sex, height and weight using a validated Chinese anthropometric equation; height/weight were measured with Seca 213 and Omron HN-286 devices.	Among older Chinese adults, WW and RA were associated with comparably lower odds of sarcopenia. Inactive = reference. WW OR 0.62 (0.42–0.94); RA OR 0.63 (0.53–0.74); Direct WW vs. RA: not reported. Possible sarcopenia, WW OR 0.58 (95% CI: 0.37–0.89); Confirmed sarcopenia, WW OR 0.99 (95% CI: 0.44–2.22).
Y. Wang et al. [29]	BMD	Age- and sex-specific Z-scores were calculated. Low BMD was present if any assessed site had a Z-score < −1.0. Femoral sites: total femur, femoral neck, trochanter and intertrochanter; lumbar BMD was the mean of L1–L4.	Femoral and lumbar-spine BMD were measured by DXA under NHANES quality control. Femoral reference values used White participants from NHANES III; lumbar reference values used same-age White participants from the study cycles.	Compared with inactive adults, those following the WW pattern had lower odds of low BMD, with associations comparable to those observed in the RA group. Inactive = reference. WW: OR 0.65 (0.46–0.92). RA: OR 0.76 (0.62–0.93); Direct WW vs. RA: OR 0.85 (0.59–1.23).
Xu et al. [30]	Osteoporosis	WHO criterion: osteoporosis = BMD at any femoral region at least 2.5 SD below the young-adult reference mean.	Femoral BMD was measured by DXA using a Hologic QDR-4500A fan-beam densitometer.	Adults aged ≥50 years who achieve the WHO-recommended 150-min weekly activity target A within 1–2 days per week exhibit a significant association with reduction in OP risk compared to IA Inactive = reference. WW: OR 0.11 (0.03–0.36); RA: OR 0.69 (0.47–1.00); Direct WW vs. RA: not reported. Total Femur Bone BMD/Femoral Neck BMD/Trochanter BMD/Intertrochanteric region BMD: WW: β 0.049 (0.007–0.090)/0.050(0.010–0.097)/0.041 (0.003–0.079)/0.048 (0.001–0.094); RA: β 0.007 (−0.014–0.029)/0.008 (−0.010–0.026)/0.006 (−0.014–0.026)/0.011 (−0.012–0.034). Direct WW vs. RA: not reported. Additional outcomes, including gout and musculotendinous and soft tissue disorders, are reported in Supplementary Table S4.
Zhu et al. [31]	Musculoskeletal disorders	Categories based on ICD-10 codes; OA: M15–M19; inflammatory arthritis: M05–M07/M45; spinal degenerative changes: M47–M48/M50–M51/M54; gout: M10; osteoporosis: M80–M82; musculotendinous/soft-tissue disorders: M60–M79.	Outcomes were ascertained through the UK Biobank First Occurrences registry, which combines primary care, hospital inpatient, death-registry and baseline self-reported data.	Relative to inactive adults, both RA and WW patterns were associated with lower risks of OA, SDCs, and osteoporosis. Inactive = reference; RA/WW HR OA: 0.76 (0.69–0.83)/0.83 (0.77–0.89); InfA: 0.79 (0.58–1.08)/0.75 (0.58–0.98); spinal degenerative changes 0.85 (0.76–0.95)/0.85 (0.78–0.94); osteoporosis 0.74 (0.62–0.88)/0.75 (0.65–0.87); Direct WW vs. RA: OA: 1.11 (1.02, 1.20); InfA: 0.98 (0.73, 1.31); SDCs: 1.00 (0.90, 1.11); osteoporosis: 1.00 (0.84, 1.18).

Notes: Effect estimates are from the most fully adjusted primary model and compare RA or WW with inactive participants unless otherwise stated. Inactive is written in full because IA denotes insufficiently active in some studies. ASM, appendicular skeletal muscle mass; BMD, bone mineral density; DXA, dual-energy X-ray absorptiometry; eASM, estimated appendicular skeletal muscle mass; EWGSOP2, European Working Group on Sarcopenia in Older People 2; FDR, false discovery rate; FNIH, Foundation for the National Institutes of Health; HR, hazard ratio; MSM, musculoskeletal multimorbidity; InfA, inflammatory arthritis; OA, osteoarthritis; OR, odds ratio; RA, regularly active; SDC, spinal degenerative changes; WW, weekend warrior.

**Table 4:** NOS appraisal of the included cohort studies.

Study	Year	Selection	Comparability	Outcome	Total
Cohort studies (*n* = 4)					
Huang et al. [24]	2026	****	**	***	9
Liu et al. [27]	2026	****	**	***	9
Song et al. [28]	2025	****	**	**	8
Zhu et al. [31]	2026	****	**	***	9

Note: The number of asterisks denotes the quality rating: low (0–3 stars), moderate (4–6 stars), and high (7–9 stars).

**Table 5:** AHRQ-based appraisal of the included cross-sectional studies.

Study	Year	1	2	3	4	5	6	7	8	9	10	11	Total
Cross-sectional studies (*n* = 6)													
Kim et al. [22]	2026	1	1	1	1	1	1	1	1	0	0	1	9
H. Li & Wang [25]	2025	1	1	1	1	1	1	1	1	1	0	1	10
Z. Li et al. [26]	2025	1	1	1	1	1	1	1	1	0	0	1	9
S. Wang et al. [23]	2026	1	1	1	1	1	1	1	1	0	0	1	9
Y. Wang et al. [29]	2025	1	1	1	1	1	1	1	1	0	0	1	9
Xu et al. [30]	2025	1	1	1	1	1	1	1	1	0	0	1	9

## Data Availability

No new data were created or analyzed in this study.

## Supplementary Materials

The following supporting information can be downloaded at: https://www.mdpi.com/article/10.3390/healthcare14213642/s1. Table S1: Operational definitions and classification criteria of the weekend warrior physical activity pattern across the included studies. Table S2: Database search strategies. Table S3: Methodological quality assessment tools. Table S4: Associations of physical activity patterns with gout and musculotendinous and soft tissue disorders.
