# Supplementary material for: Association Between the “Weekend Warrior” Physical Activity Pattern and Musculoskeletal Health Outcomes: A Systematic Review
Source: Healthcare (Basel). 2026 Oct 27;14(21):3642. doi: 10.3390/healthcare14213642 (PMC13763135; doi:10.3390/healthcare14213642)
Supplement: Supplementary file 1 [file healthcare-14-03642-s001.zip › Table S1.pdf]

Table S1. Criteria used to define the weekend warrior across included studies.

Author	Guideline-Based Threshold	Number of Days/Sessions	Days/Sessions Proportion Requirement	Exact Definition of WW	Definition of Inactive/Insufficiently Active
Huang et al. (2026)	≥150 min MVPA/week	1–2 days/week	>50%	Participants who met the MVPA threshold and accumulated >50% of their total weekly MVPA within 1–2 days were defined as having a weekend warrior pattern.	Inactive: accelerometer-derived MVPA <150 min/week.
Kim et al. (2026)	≥150 min/week MPA, or ≥75 min/week VPA, or ≥150 min/week MVPA	1–2 days/week	NA	According to whether or not they met the World Health Organization recommendations of ≥150 minute per week for MPA, ≥75 minute per week VPA, or ≥150 minute per week for MVPA. Participants who engaged in PA on 1 to 2 days per week were classified as the WW group.	Inactive: did not meet any of the reported leisure-time PA criteria (MPA ≥150 min/week, VPA ≥75 min/week, or MVPA ≥150 min/week).
H. Li & Wang. (2025)	≥150 min total PA/week	1–2 sessions/week	NA	Weekend warrior (total minutes of PA ≥150 min per week, performed in one or two sessions).	Inactive: no reported moderate or vigorous PA.<br>Insufficiently active: total PA <150 min/week.<br>Total PA = MPA minutes + 2 × VPA minutes.

Liu et al. (2026)	≥150 min MVPA/week	1–2 days/week	≥50%	For our primary analyses, we defined active status as the WHO physical activity guideline-based threshold (≥150 min/week of MVPA) with ≥50% of total MVPA over 1–2 days.	Inactive: MVPA <150 min/week.
Z. Li et al. (2025)	≥150 min total PA/week	< 2 sessions/week	NA	"Weekend warriors" was defined as individuals who meet weekly physical activity guidelines (≥150 minutes) but exercise infrequently (<2 sessions/week).	Inactive: total recreational PA <150 min/week.<br>Total PA = MPA minutes + 2 × VPA minutes.
Song et al. (2025)	≥150 min MVPA/week	1–2 days/week	≥50%	Active weekend warrior (≥150 min of MVPA per week and ≥50% of total MVPA concentrated within 1–2 days [whether or not it is on the weekend]).	Inactive: MVPA <150.0 min/week in the guideline-based analysis.
S. Wang et al. (2026)	≥150 min MVPA/week	≤ 2 sessions/week	NA	Weekend warrior (≥150 MVPA minutes/week with ≤2 sessions/week).	Inactive: no MVPA.<br>Insufficiently active (IA): MVPA <150 min/week.<br>Weekly MVPA = MPA minutes + 2 × VPA minutes.
Y. Wang et al. (2025)	≥150 min total PA/week	1–2 sessions/week	NA	Sufficiently active, participants reported total PA ≥150 min in one or two sessions per week.	Inactive: total PA = 0 min/week.<br>Insufficiently active: total PA <150 min/week.

					Total PA = MPA minutes + 2 × VPA minutes.
Xu et al. (2025)	≥150 min/week MPA or ≥75 min/week VPA	1–2 sessions/week	NA	Weekend warrior: reporting at least 150 min/week in moderate-intensity or at least 75 min/week in vigorous-intensity activities from 1 or 2 sessions.	Inactive: no reported moderate or vigorous PA, or both MPA <150 min/week and VPA <75 min/week.
Zhu et al. (2026)	≥150 min MVPA/week	Most active 1–2 days/week	≥50%	Weekend warrior (≥150 min/week with ≥50% of weekly MVPA accrued within the most active 1–2 days).	Inactive: MVPA <150 min/week.

Note: MPA, moderate-intensity physical activity; VPA, vigorous-intensity physical activity; MVPA, moderate-to-vigorous physical activity
