# Supplementary material for: Association Between the “Weekend Warrior” Physical Activity Pattern and Musculoskeletal Health Outcomes: A Systematic Review
Source: Healthcare (Basel). 2026 Oct 27;14(21):3642. doi: 10.3390/healthcare14213642 (PMC13763135; doi:10.3390/healthcare14213642)
Supplement: Supplementary file 1 [file healthcare-14-03642-s001.zip › Table S2.pdf]

Table S2 Database search strategies

Database and platform	Search fields	Search strategy	Limits and last search
PubMed	Untagged search; no explicit field restriction	("weekend warrior" OR "weekend warriors" OR "WW") AND ("musculoskeletal system" OR "osteoporosis" OR "sarcopenia" OR "osteoarthritis" OR "bone mineral density" OR "BMD" OR "bone health" OR "musculoskeletal disorder")	English<br>30 June 2026
Web of Science	Topic (TS): title, abstract, author keywords, and Keywords Plus	TS=("weekend warrior" OR "weekend warriors" OR "WW") AND TS=("musculoskeletal system" OR "osteoporosis" OR "sarcopenia" OR "osteoarthritis" OR "bone mineral density" OR "BMD" OR "bone health" OR "musculoskeletal disorder")	English<br>30 June 2026
Scopus	Title, abstract, and keywords (TITLE-ABS-KEY)	TITLE-ABS-KEY(("weekend warrior" OR "weekend warriors" OR "WW") AND ("musculoskeletal system" OR "osteoporosis" OR "sarcopenia" OR "osteoarthritis" OR "bone mineral density" OR "BMD" OR "bone health" OR "musculoskeletal disorder"))	English<br>30 June 2026

Note: All databases were searched from inception to 30 June 2026. Strategies are presented in database-specific syntax using the reported keywords and fields. No controlled vocabulary terms were explicitly entered. WW, weekend warrior; BMD, bone mineral density.
