# Supplementary material for: Association Between the “Weekend Warrior” Physical Activity Pattern and Musculoskeletal Health Outcomes: A Systematic Review
Source: Healthcare (Basel). 2026 Oct 27;14(21):3642. doi: 10.3390/healthcare14213642 (PMC13763135; doi:10.3390/healthcare14213642)
Supplement: Supplementary file 1 [file healthcare-14-03642-s001.zip › Table S3.pdf]

Supplementary Table S3 (a). Newcastle–Ottawa Scale (NOS) item key and cohort study appraisal

Criterion	Basis for a star
Selection S1	Representativeness of the exposed cohort
Selection S2	Comparison cohort drawn from the same source population
Selection S3	Reliability/ascertainment of physical-activity exposure
Selection S4	Outcome absent at baseline
Comparability C1	Control of the most important confounder(s), as prespecified
Comparability C2	Control of additional relevant confounders
Outcome O1	Outcome measurement/ascertainment quality
Outcome O2	Follow-up duration adequate for the endpoint
Outcome O3	Completeness and handling of loss to follow-up

Supplementary Table S3 (b). 11-item Agency for Healthcare Research and Quality (AHRQ) item key and cross-sectional study appraisal

Item	AHRQ Cross-Sectional Study Quality item
1	Source of the information: survey, database or record review
2	Inclusion and exclusion criteria, or an explicit reference to them
3	Period during which participants were identified
4	Consecutive enrollment, if the source is not population-based
5	Masking for assessors of subjective aspects of the study
6	Procedures used for measurement or data quality assurance
7	Reasons for any exclusion from the analysed sample
8	Identification, assessment and/or adjustment of confounding
9	Handling of missing values, when applicable
10	Response rates and completeness of collected data
11	Expected follow-up and extent of incomplete data/follow-up, if any
