# Supplementary material for: Association Between the “Weekend Warrior” Physical Activity Pattern and Musculoskeletal Health Outcomes: A Systematic Review
Source: Healthcare (Basel). 2026 Oct 27;14(21):3642. doi: 10.3390/healthcare14213642 (PMC13763135; doi:10.3390/healthcare14213642)
Supplement: Supplementary file 1 [file healthcare-14-03642-s001.zip › Table S4.pdf]

Supplementary Table S4. Associations of physical activity patterns with gout and musculotendinous and soft tissue disorders.

Outcome	RA vs. inactive HR (95% CI)	WW vs. inactive HR (95% CI)	WW vs. RA HR (95% CI)
Gout	0.61 (0.45–0.83)	0.85 (0.68–1.07)	1.47 (1.11–1.94)
Musculotendinous and soft tissue disorders	0.92 (0.82–1.02)	0.95 (0.87–1.04)	1.04 (0.95–1.14)

Note. RA, regularly active; WW, weekend warrior; HR, hazard ratio; CI, confidence interval.
